# Supplementary material for: A Comparison of Vaccine Hesitancy of COVID-19 Vaccination in China and the United States
Source: Vaccines (Basel). 2021 Jun 14;9(6):649. doi: 10.3390/vaccines9060649 (PMC8232230; doi:10.3390/vaccines9060649)
Supplement: Supplementary file 1 [file vaccines-09-00649-s001.zip › vaccines-1221885-supplementary.pdf]

Supplementary information

Table of Contents

Fig S1. COVID-19 vaccination acceptance comparison between China and the US depending on sex and age intervals after propensity score matching 2

Fig S2. COVID-19 vaccination acceptance comparison between China and the US depending on education and annual income after propensity score matching 3

Fig S3. COVID-19 vaccination acceptance comparison between China and the US depending on occupation after propensity score matching 4

Fig S4. COVID-19 vaccination acceptance in China before propensity score matching 5

Fig S5. COVID-19 vaccination acceptance in the US before propensity score matching 7

Fig S6. COVID-19 vaccination preference comparison between China and the US depending on sex after propensity score matching 9

Table S1. Behavioral and psychological results 10

Table S3. The Pearson Correlation matrix between the baseline characteristics and acceptance 13

Fig S1. COVID-19 vaccination acceptance comparison between China and the US depending on sex and age intervals after propensity score matching

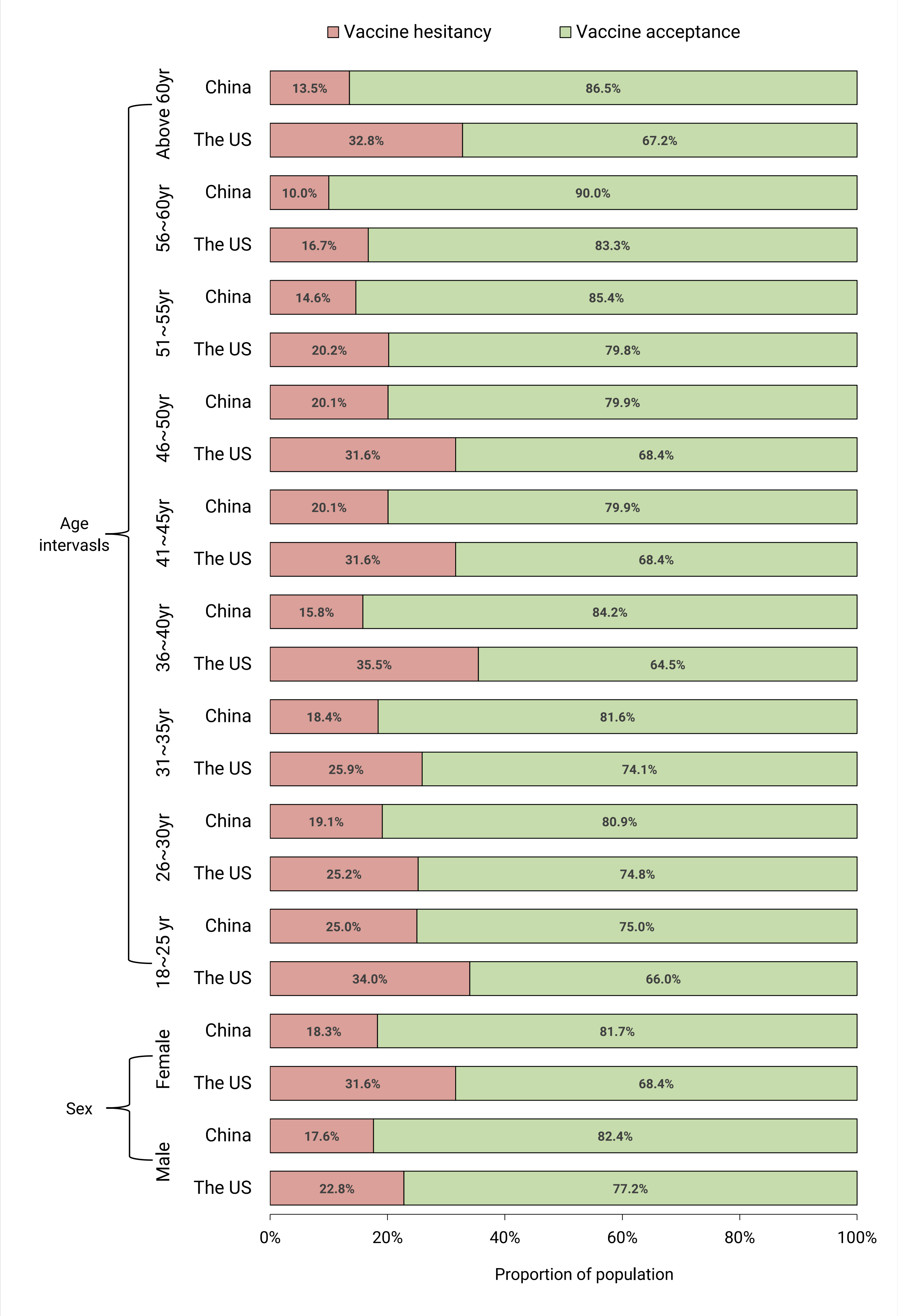

Fig S2. COVID-19 vaccination acceptance comparison between China and the US depending on education and annual income after propensity score matching

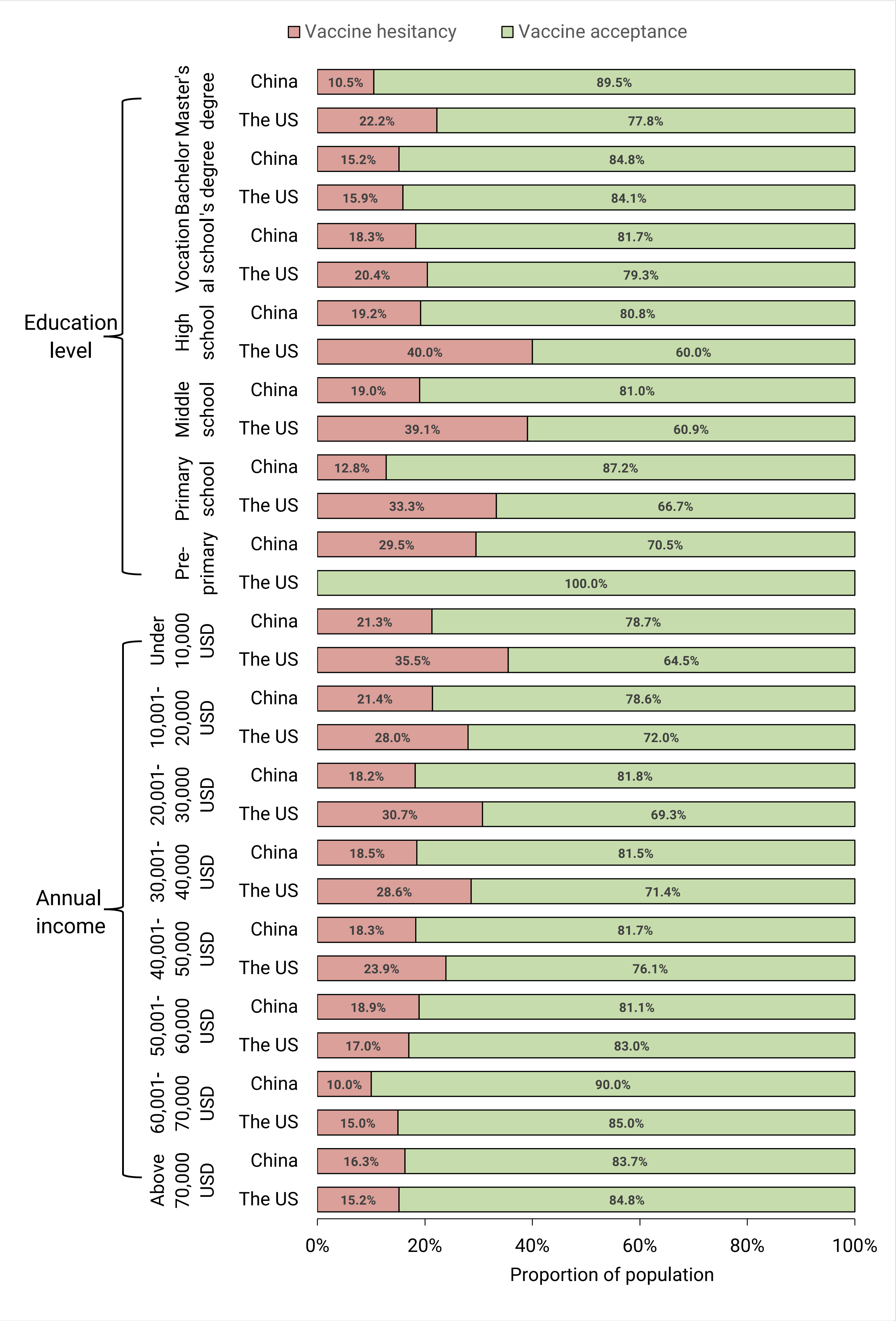

Fig S3. COVID-19 vaccination acceptance comparison between China and the US depending on occupation after propensity score matching

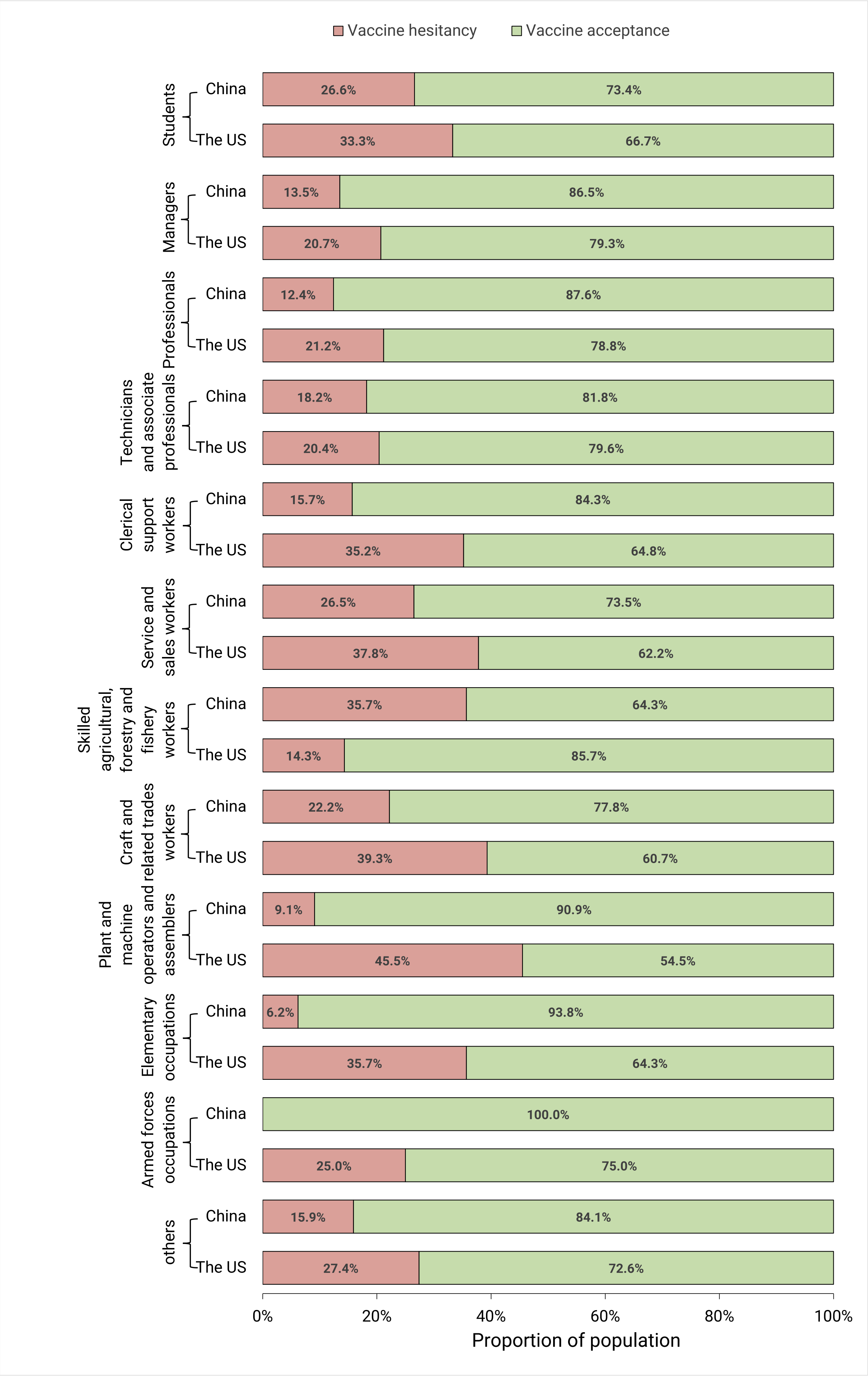

Fig S4. COVID-19 vaccination acceptance in China before propensity score matching

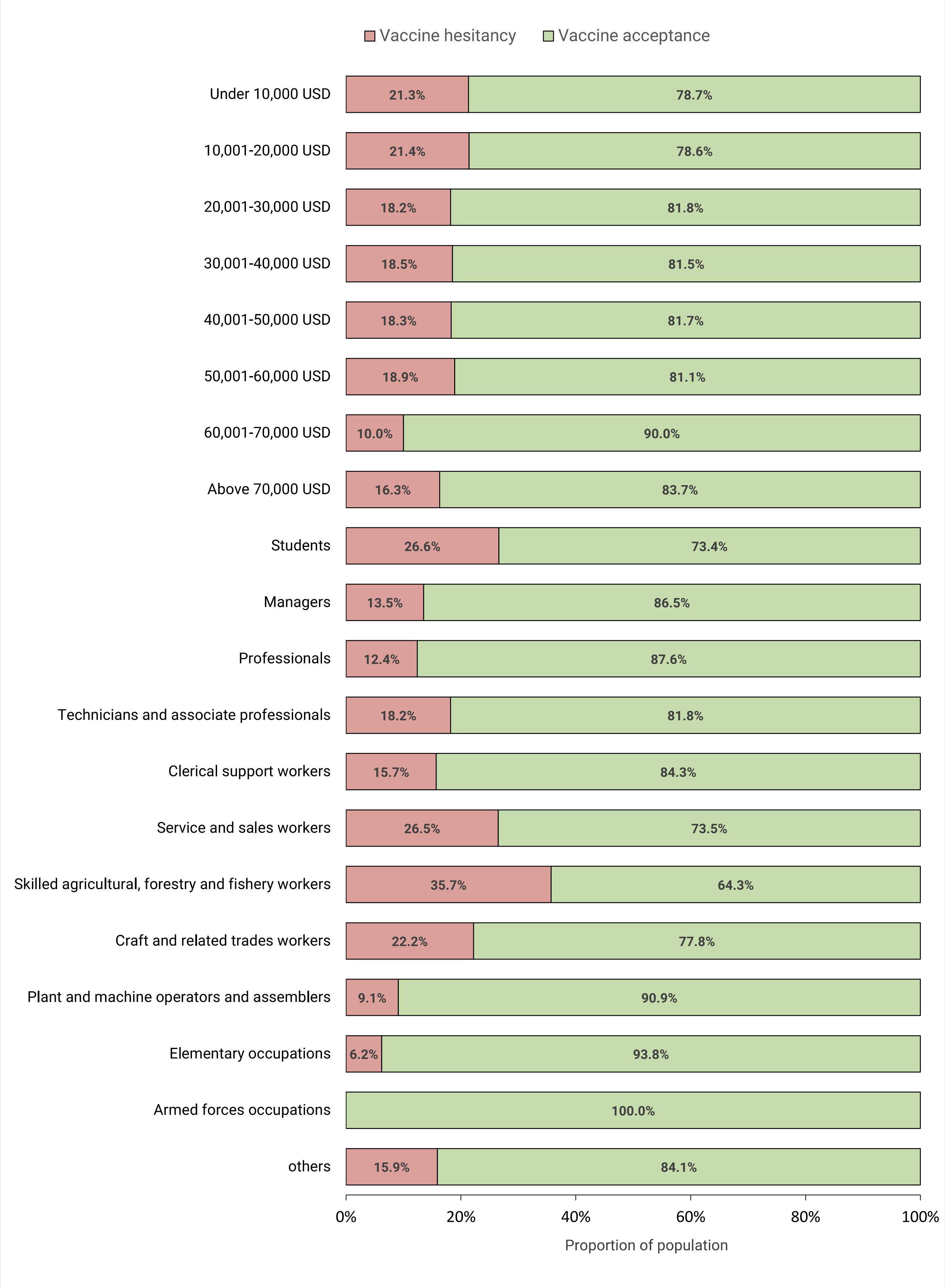

(continued)

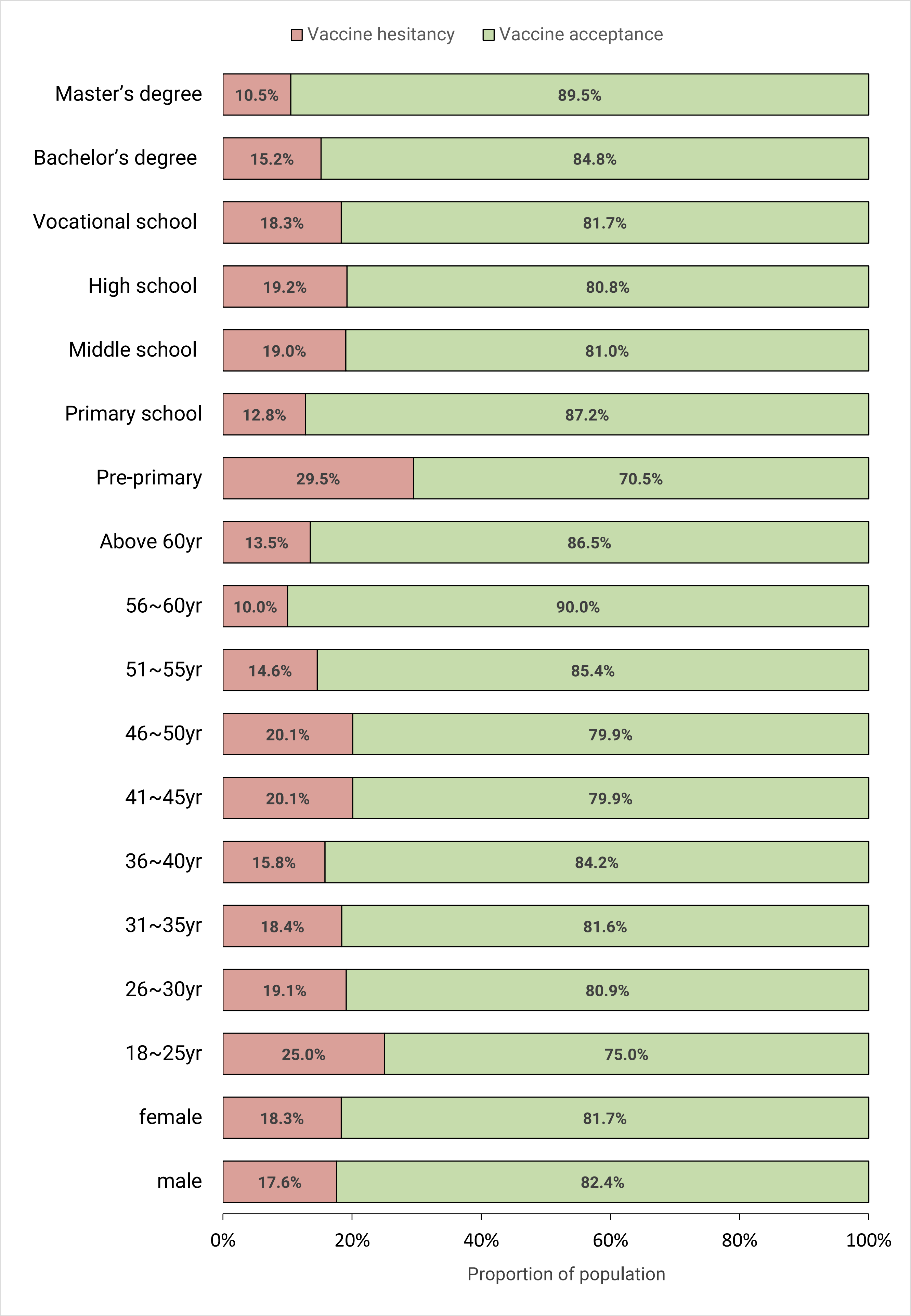

Vaccine hesitancy
Vaccine acceptance
Master's degree 10.5% 89.5%
Bachelor's degree 15.2% 84.8%
Vocational school 18.3% 81.7%
High school 19.2% 80.8%
Middle school 19.0% 81.0%
Primary school 12.8% 87.2%
Pre-primary 29.5% 70.5%
Above 60yr 13.5% 86.5%
56~60yr 10.0% 90.0%
51~55yr 14.6% 85.4%
46~50yr 20.1% 79.9%
41~45yr 20.1% 79.9%
36~40yr 15.8% 84.2%
31~35yr 18.4% 81.6%
26~30yr 19.1% 80.9%
18~25yr 25.0% 75.0%
female 18.3% 81.7%
male 17.6% 82.4%
0%
20%
40%
60%
80%
100%
Proportion of population

Fig S5. COVID-19 vaccination acceptance in the US before propensity score matching

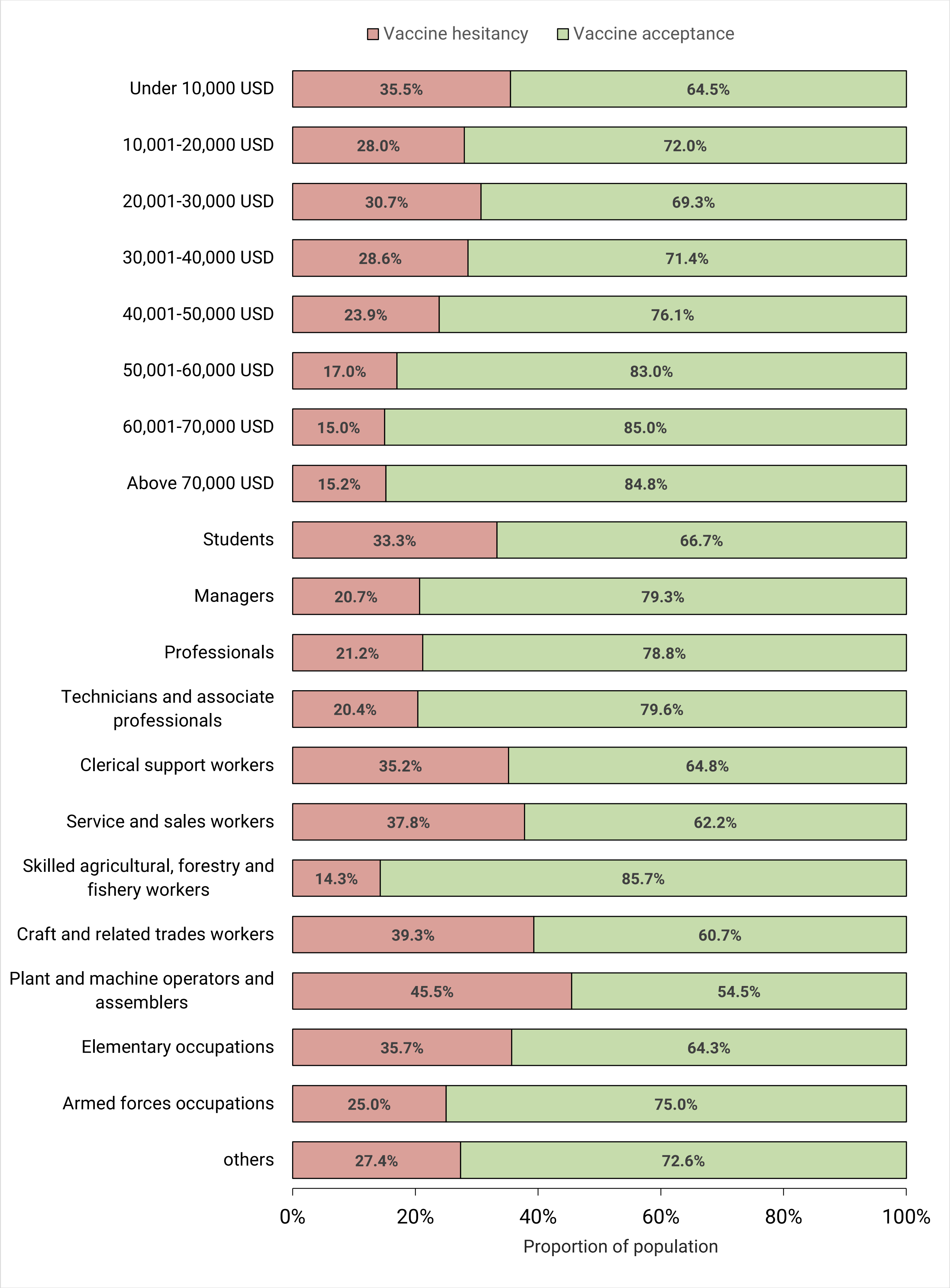

(Continued)

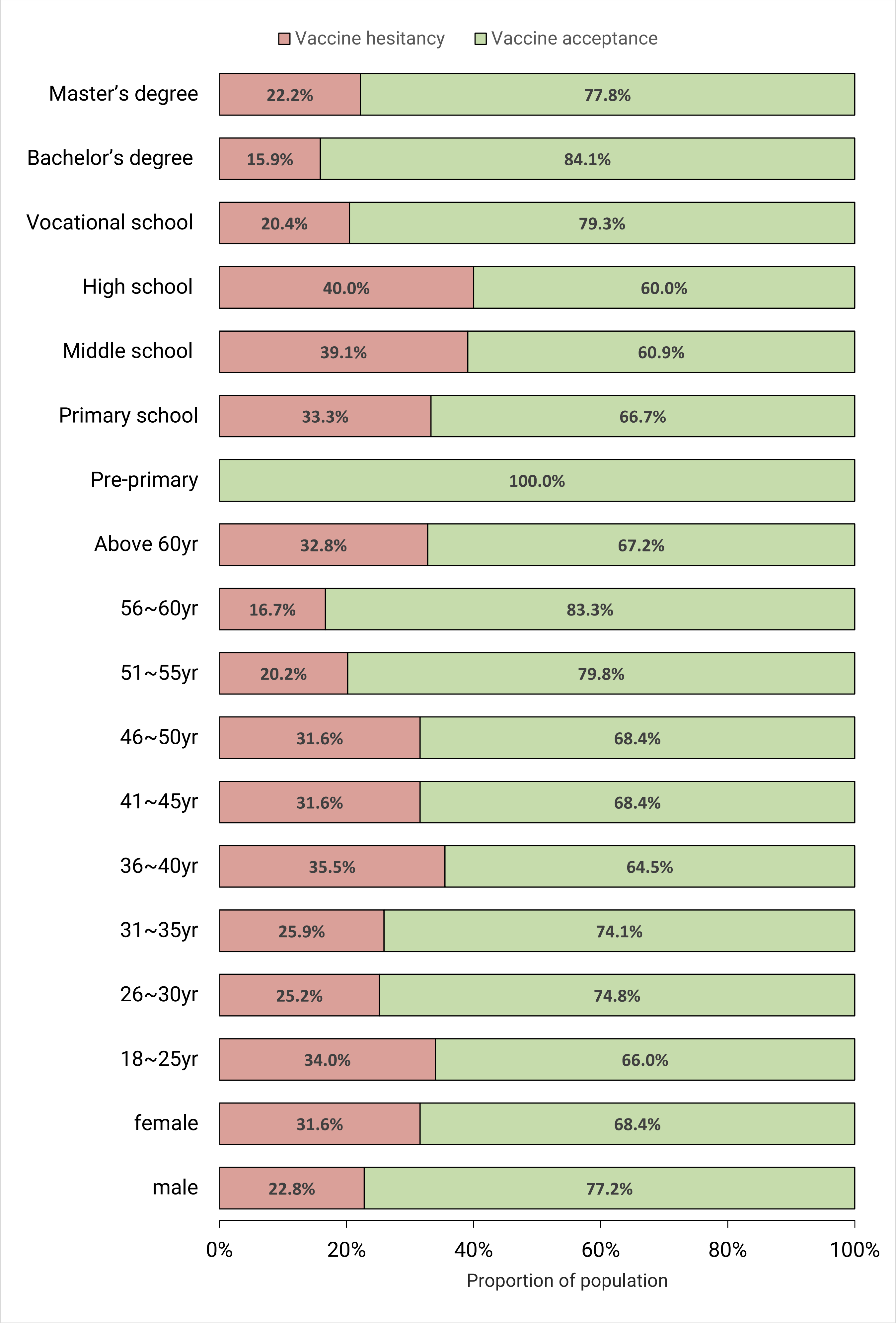

Vaccine hesitancy
Vaccine acceptance
Master’s degree
22.2%
77.8%
Bachelor’s degree
15.9%
84.1%
Vocational school
20.4%
79.3%
High school
40.0%
60.0%
Middle school
39.1%
60.9%
Primary school
33.3%
66.7%
Pre-primary
100.0%
Above 60yr
32.8%
67.2%
56~60yr
16.7%
83.3%
51~55yr
20.2%
79.8%
46~50yr
31.6%
68.4%
41~45yr
31.6%
68.4%
36~40yr
35.5%
64.5%
31~35yr
25.9%
74.1%
26~30yr
25.2%
74.8%
18~25yr
34.0%
66.0%
female
31.6%
68.4%
male
22.8%
77.2%
0%
20%
40%
60%
80%
100%
Proportion of population

Fig S6. COVID-19 vaccination preference comparison between China and the US depending on sex after propensity score matching

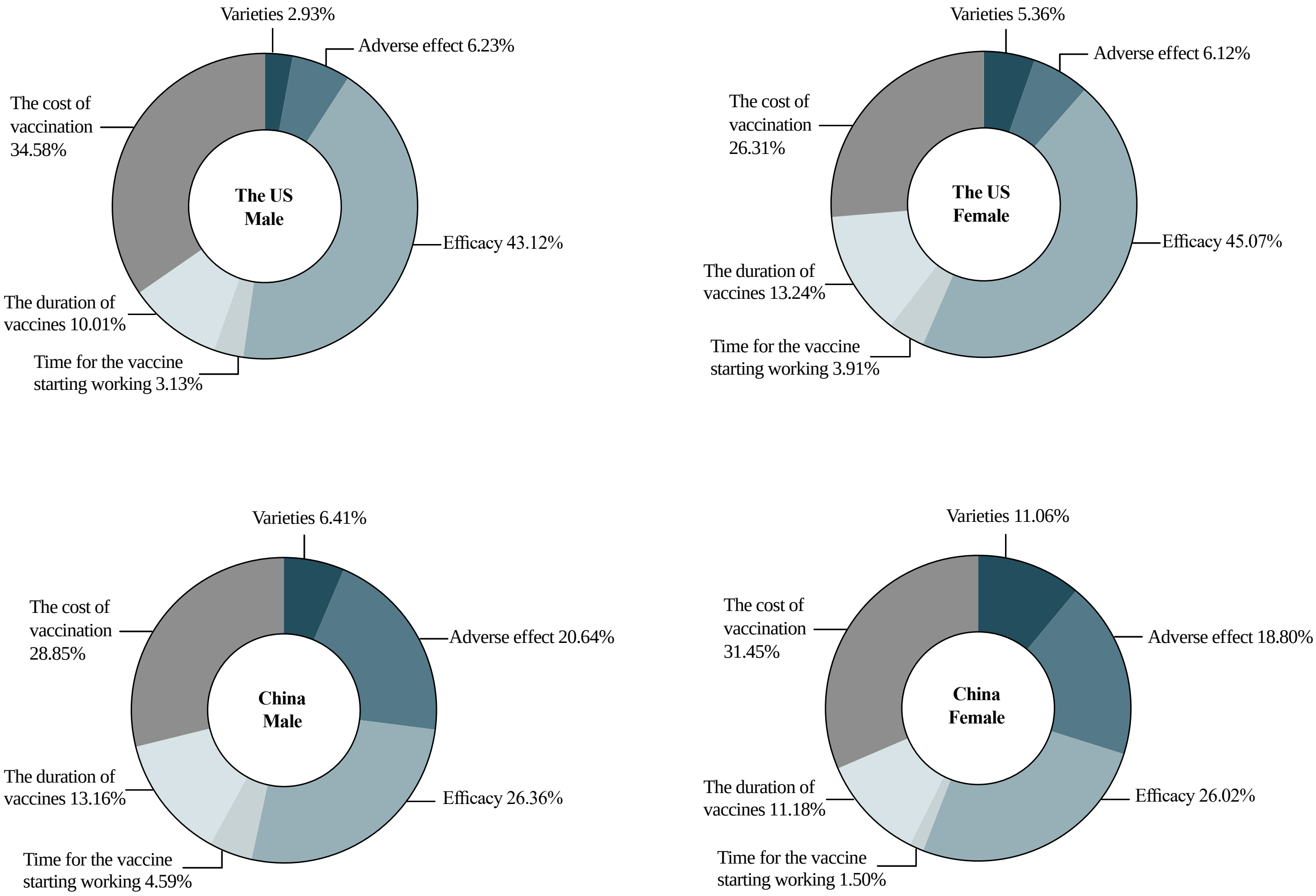

Table S1. Behavioral and psychological results

Items		Pre-PSM			Post-PSM		
		China	The US	*P* value	China	The US	*P* value
Past immunization behaviour/ adverse events		(N=5374)	(N=3701)		(N=1240)	(N=1240)	
"I have delayed getting a vaccine shot once (or more than once) for reasons other than illness or allergy."	Yes	567 (10.6%)	1246 (33.7%)	<0.001	152 (12.3%)	475 (38.3%)	<0.001
	No	4807 (89.4%)	2455 (66.3%)		1088 (87.7%)	765 (61.7%)	
"I have once decided not to get a shot of vaccine for reasons other than illness or allergy?"	Yes	528 (9.8%)	1273 (34.4%)	<0.001	124 (10.0%)	477 (38.5%)	<0.001
	No	4846 (90.2%)	2428 (65.6%)		1116 (90.0%)	763 (61.5%)	
"I have events in the past that discouraged me from getting a vaccine(s) for myself or my families?"	Yes	363 (6.8%)	1000 (27.0%)	<0.001	93 (7.5%)	375 (30.2%)	<0.001
	No	5011 (93.2%)	2701 (73.0%)		1147 (92.5%)	865 (69.8%)	
Did you ever experience an AEFI (adverse event following immunization)?	Yes	580 (10.8%)	752 (20.4%)	<0.001	148 (12.0%)	252 (20.3%)	<0.001
	No	4776 (89.2%)	2937 (79.6%)		1089 (88.0%)	987 (79.7%)	
Cues to action							
"I am recommended by a doctor to get COVID-19 vaccination."	Yes	1763 (32.8%)	1968 (53.2%)	<0.001	395 (31.9%)	622 (50.2%)	<0.001
	No	3611 (67.2%)	1733 (46.8%)		845 (68.1%)	618 (49.8%)	
"I am recommended by the local health board to get COVID-19 vaccination."	Yes	1995 (37.1%)	2312 (62.5%)	<0.001	444 (35.8%)	736 (59.4%)	<0.001
	No	3379 (62.9%)	1389 (37.5%)		796 (64.2%)	504 (40.6%)	
"I heard that my friends/families are being vaccinated."	Yes	2370 (44.1%)	2572 (69.5%)	<0.001	502 (40.5%)	803 (64.8%)	<0.001
	No	3004 (55.9%)	1129 (30.5%)		738 (59.5%)	437 (35.2%)	
**Likert Scale**							
**Perceived knowledge (source of information)**							
"I feel I get enough information about COVID-19 vaccines and their safety."		5.1 (1.7)	5.1 (1.8)	0.36	5.2 (1.6)	5.0 (1.9)	0.002
"The information I receive about COVID-19 vaccines from the vaccine program is reliable and trustworthy."		5.3 (1.6)	5.0 (1.9)	<0.001	5.3 (1.5)	4.9 (1.9)	<0.001
"I trust the information I receive about COVID-19 vaccines."		5.2 (1.6)	4.1 (2.0)	<0.001	5.3 (1.6)	4.1 (2.0)	<0.001
**Perceived severity**							
"I think there is a great chance for me to be infected with COVID-19."		3.3 (2.2)	4.5 (1.8)	<0.001	3.6 (2.2)	4.5 (1.8)	<0.001
"I think a COVID-19 infection would be a serious threat to health."		5.8 (1.6)	5.1 (1.9)	<0.001	5.8 (1.6)	5.1 (1.8)	<0.001
**Perceived benefits**							
"Getting COVID-19 vaccines is a good way to protect myself from COVID-19."		5.7 (1.5)	5.4 (1.9)	<0.001	5.7 (1.4)	5.2 (2.0)	<0.001
"Getting COVID-19 vaccines is a good way to prevent COVID-19 infection spread by patients."		5.5 (1.6)	5.4 (1.8)	<0.001	5.6 (1.5)	5.3 (1.9)	<0.001
"Getting COVID-19 vaccines is a good way to protect my friends or families from infected by COVID-19."		5.7 (1.4)	5.4 (1.8)	<0.001	5.7 (1.4)	5.3 (1.9)	<0.001
**Perceived risks and barriers**							
"I am afraid that COVID-19 vaccine may, in fact, cause me to get infected with COVID-19."		3.9 (2.1)	3.0 (2.1)	<0.001	4.1 (2.1)	3.2 (2.1)	<0.001

"I am concerned that I might have a serious side effect from a shot of COVID-19 vaccine."	4.5 (1.9)	4.0 (2.1)	<0.001	4.6 (1.8)	4.2 (2.1)	<0.001
"I am concerned that the production, storage, transportation, and unprofessional injection administration may cause the COVID-19 vaccines to be unsafe for taking."	4.5 (1.9)	3.8 (2.1)	<0.001	4.6 (1.9)	3.9 (2.1)	<0.001
**Perceived safety and efficacy of vaccines**						
"I am concerned that taking the vaccines might not prevent COVID-19."	4.5 (1.8)	4.0 (2.0)	<0.001	4.5 (1.8)	4.2 (2.0)	<0.001
"It is better to develop immunity by getting vaccinated than by getting infected with COVID-19."	5.5 (1.5)	5.0 (2.0)	<0.001	5.6 (1.5)	4.9 (2.1)	<0.001
"I believe the COVID-19 vaccine is safe."	5.3 (1.5)	5.0 (1.9)	<0.001	5.3 (1.4)	4.8 (2.0)	<0.001
**General attitudes and trust for vaccines**						
"In general, prevention is better than cure."	5.9 (1.4)	5.4 (1.7)	<0.001	5.9 (1.3)	5.3 (1.7)	<0.001
"In general, vaccination is effective in preventing diseases."	5.7 (1.4)	5.6 (1.6)	0.033	5.7 (1.3)	5.5 (1.6)	<0.001
**Socio-cultural factors**						
"I am able to openly discuss my concerns about COVID-19 vaccine shots with my doctor."	5.4 (1.5)	5.0 (1.9)	<0.001	5.4 (1.4)	4.9 (1.9)	<0.001
"I feel confident that the health center or doctor's office will have the COVID-19 vaccine when I need to take it."	5.5 (1.5)	5.5 (1.7)	0.41	5.5 (1.5)	5.4 (1.7)	0.004
"I trust that my government is making decisions in my best interest with respect to what COVID-19 vaccines are provided."	5.4 (1.6)	4.6 (1.9)	<0.001	5.4 (1.6)	4.5 (1.9)	<0.001
"I think COVID-19 vaccines are more important for boys/men" or "I think COVID-19 vaccines are more important for girls/women."	5.7 (1.4)	4.5 (1.9)	<0.001	5.7 (1.4)	4.4 (2.0)	<0.001
"I decide to take/not to take the COVID-19 vaccine because of religious or cultural reasons."	4.0 (2.2)	3.1 (2.1)	<0.001	4.2 (2.1)	3.1 (2.0)	<0.001
"I believe that people are risking their health or the health of the society if they do not take a COVID-19 vaccine."	3.4 (2.3)	2.4 (2.0)	<0.001	3.5 (2.3)	2.5 (2.1)	<0.001
"I think it is important for everyone to get the recommended COVID-19 vaccines for themselves."	5.3 (1.6)	4.9 (2.0)	<0.001	5.3 (1.6)	4.7 (2.1)	<0.001
"It's important for me to spend more than one hour in travel time to get a COVID-19 vaccine."	5.7 (1.4)	5.1 (2.0)	<0.001	5.7 (1.4)	4.9 (2.0)	<0.001
"I believe that COVID-19 vaccine producers care about my health more than their profit."	5.7 (1.5)	3.6 (2.1)	<0.001	5.7 (1.5)	3.5 (2.2)	<0.001

Table S2. The attributes and levels of vaccine acceptance and preference.

Attributes	Attributes' description	Levels
Vaccine varieties	Different varieties of vaccines developed in different countries	mRNA; adenovirus vector vaccines; Inactivated vaccine
Adverse effect	Extent of adverse effect after getting vaccinated	very mild; mild; moderate
Efficacy	The efficacy of vaccines that protect the vaccinators from getting infected with COVID-19	55%; 65%; 75%; 85%; 95%
Time for the vaccine starting working	Time period from getting vaccinated to the vaccine starts working	5 days; 10 days; 15 days; 20 days
The duration of vaccine works	Time period from the vaccines starting to work to its invalidation	5 months; 10 months; 15 months; 20 months
The cost of vaccination	The cost of whole vaccination process	$0; $50; $100; $150; $200

Table S3. The Pearson Correlation matrix between the baseline characteristics and acceptance

Variables	-1	-2	-3	-4	-5	-6	-7	-8	-9	-10	-11	-12	-13	-14	-15	-16	-17	-18	-19	-20	-21	-22	-23	-24	-25	-26
(1) country	1																									
(2) acceptance	0.076*	1																								
(3) acceptance under social cues	0.094*	0.783*	1																							
(4) sex	0.031*	-0.061*	-0.047*	1																						
(5) age	0.061*	0.02	0.013	-0.026	1																					
(6) education	-0.264*	0.062*	0.051*	-0.004	-0.264*	1																				
(7) occupation	-0.099*	-0.049*	-0.045*	0.039*	0.321*	-0.286*	1																			
(8) Annual earning	-0.560*	0.018	-0.003	-0.098*	0.017	0.420*	-0.119*	1																		
(9) ever_infected with COVID-19	0.264*	-0.025	-0.011	0.050*	0.051*	-0.109*	0.014	-0.144*	1																	
(10) acquaintance ever infected with COVID-19	0.690*	0.015	0.02	-0.019	0.026	-0.193*	-0.060*	-0.414*	0.383*	1																
(11) marital status	0.028*	-0.043*	-0.051*	0.014	0.378*	-0.120*	0.189*	0.062*	0.064*	0.066*	1															
(12) source_health~r	-0.007	0.066*	0.068*	0.014	-0.051*	0.096*	-0.053*	0.039*	-0.036*	-0.026	-0.041*	1														
(13) source_PH_dep~t	-0.069*	0.068*	0.080*	0.065*	-0.022	0.088*	-0.024	0.077*	0.016	-0.106*	-0.053*	0.270*	1													
(14) source_news	0.102*	0.068*	0.074*	0.059*	0.033*	0.003	0.007	-0.047*	0.089*	0.039*	-0.063*	-0.015	0.098*	1												

(15) source_social~a	0.102*	0.036*	0.042*	0.016	-0.161*	0.047*	-0.082*	-0.063*	-0.008	0.057*	-0.085*	0.080*	0.069*	0.156*	1											
(16) source_friend~y	0.062*	0.022	0.034*	0.044*	0.013	-0.043*	0.026	-0.049*	0.022	0.036*	0.018	0.107*	0.069*	0.126*	0.327*	1										
(17) source_Employer	-0.075*	0.026	0.027*	-0.022	-0.011	0.061*	0.004	0.110*	-0.033*	-0.068*	0.036*	0.139*	0.099*	0.004	0.105*	0.191*	1									
(18) source_ad	0.097*	0.033*	0.023	-0.018	0.022	-0.033*	0.001	-0.059*	0.036*	0.069*	0.026	0.210*	0.160*	0.092*	0.179*	0.226*	0.291*	1								
(19) source_other	-0.068*	-0.043*	-0.059*	-0.011	-0.001	0.009	0.063*	0.032*	-0.028*	-0.033*	-0.001	-0.042*	-0.023	-0.079*	-0.055*	-0.041*	-0.015	-0.006	1							
(20) delay_vaccine	0.284*	0.122*	0.133*	0.004	0.058*	-0.098*	-0.018	-0.122*	0.267*	0.231*	-0.004	-0.002	0.029*	0.076*	-0.017	-0.022	-0.061*	0	-0.033*	1						
(21) cancel_vaccine	0.303*	0.181*	0.179*	-0.011	0.062*	-0.087*	-0.02	-0.124*	0.232*	0.242*	-0.001	-0.001	0.019	0.081*	-0.004	-0.01	-0.049*	-0.002	-0.056*	0.659*	1					
(22) discrouraging~s	0.279*	0.125*	0.149*	0.013	0.045*	-0.068*	0.002	-0.128*	0.275*	0.227*	-0.024	-0.007	0.048*	0.107*	-0.027	-0.007	-0.054*	0.003	-0.028*	0.520*	0.517*	1				
(23) AEFI	0.133*	0.048*	0.035*	0.025	0.043*	-0.064*	0.014	-0.069*	0.254*	0.144*	-0.022	-0.023	-0.006	0.081*	-0.051*	-0.032*	-0.072*	-0.011	-0.004	0.392*	0.354*	0.451*	1			
(24) doctor_recomm~d	0.203*	-0.195*	-0.186*	0.045*	-0.045*	-0.104*	-0.015	-0.148*	0.165*	0.209*	-0.008	-0.128*	-0.111*	0.033*	0.02	-0.013	-0.063*	-0.029*	0.021	0.133*	0.094*	0.135*	0.196*	1		
(25) local_recommend	0.249*	-0.171*	-0.167*	0.030*	-0.039*	-0.109*	-0.026	-0.159*	0.149*	0.244*	0.006	-0.119*	-0.142*	0.025	0.027	-0.007	-0.082*	-0.023	0.008	0.139*	0.116*	0.126*	0.178*	0.744*	1	
(26) ff_vaccinated	0.251*	-0.135*	-0.142*	-0.017	-0.008	-0.183*	0.013	-0.184*	0.137*	0.283*	0.036*	-0.069*	-0.138*	-0.009	0.02	-0.063*	-0.071*	0.004	0.023	0.128*	0.125*	0.116*	0.156*	0.424*	0.454*	1
